# Assessment of socio-economic and demographic factors on child poverty: a case study of Northern Zone of Tanzania

**DOI:** 10.3389/fsoc.2026.1905874

**Published:** 2026-10-26

**Authors:** Leon Anthony Mutalemwa, Hossein Miraji

**Affiliations:** 1 Institute of Development Studies, The University of Dodoma, Dodoma, Tanzania; 2 Department of Chemistry, College of Natural and Mathematical Sciences, University of Dodoma, Dodoma, Tanzania

**Keywords:** child poverty, demographic factors, ordered logistic regression, socio-economic factors, Tanzania

## Abstract

Despite sustained national and international efforts, child poverty remains a persistent challenge in developing countries specifically Tanzania. Growing up in poverty can affect personal future wellbeing and negatively affect persons' life opportunities. This study examines the socio-economic and demographic determinants of child poverty among children aged 7–17 years in the Northern Zone of Tanzania. Using a cross-sectional survey design, data were collected from 504 children selected through a multi-stage sampling procedure in Moshi District Council and Moshi Municipal Council. The multidimensional poverty outcome variable was constructed using average indexing, whereby the three dimensions were aggregated into a continuous composite index for each child. Fixed cut-off thresholds were then applied to this composite index to segment the population into three progressive, rank-ordered categories: Very Poor (high deprivation/low average scores), Poor (moderate average scores), and Not Poor (low deprivation/high average scores). Descriptive statistics, bivariate analyses, and an ordered logistic regression model were employed to examine variations in child poverty status. The results indicate that age of the child, household size, household structure, and employment status of the household head are significant predictors of child poverty. Older children and those residing in two-parent and non-agricultural households were less likely to experience poverty, while children from large households and households with unemployed heads faced substantially higher risks. The findings underscore the importance of livelihood diversification, household-level economic stability, and family planning interventions in reducing child poverty. The study contributes to the literature by incorporating children's self-reported experiences of deprivation into the analysis of multidimensional child poverty.

## 1 Introduction

Poverty is a multidimensional phenomenon encompassing deprivations in income, health, education, nutrition, housing, and access to basic services (United Nations Development Programme, 2020). While poverty is often measured at the household level, children experience poverty differently and more acutely, particularly during school-age years when deprivation can result in irreversible losses in cognitive development, educational attainment, and future earnings (Grantham-McGregor et al., 2007; Heckman and Masterov, 2007).

Despite substantial global efforts to eradicate child poverty including the Sustainable Development Goals (SDG 1), cash transfer programs such as Tanzania's Productive Social Safety Net serving over 1.1 million households), and fee-free basic education policies across Africa but about 74% of children aged 0–17 remain multidimensionally poor (deprived in three or more dimensions; UNICEF and National Bureau of Statistics Tanzania, 2019; United Nations Children's Fund, 2023; World Bank, 2022). Child poverty warrants distinct analytical and policy attention for three key reasons. First, children aged 5–17 are especially vulnerable to long-term developmental consequences when basic needs remain unmet. Second, deprivation during childhood perpetuates intergenerational poverty through reduced human capital accumulation. Third, children lack agency to independently exit poverty, making child poverty a critical human-rights concern (UNICEF and World Bank, 2023a).

Globally, over 333 million children lived in extreme monetary poverty in 2022, with Sub-Saharan Africa accounting for more than 70 percent of this burden (UNICEF and World Bank, 2023b). In Tanzania, approximately 63 percent of children aged 5–17 experience multidimensional poverty, driven by household economic insecurity, large family sizes, and rural–urban disparities (National Bureau of Statistics, 2019). Despite growing literature on child poverty, most Tanzanian studies equate child poverty with household poverty and rarely incorporate children's own perspectives (de Milliano and Plavgo, 2018; UNICEF and National Bureau of Statistics Tanzania, 2019).

Addressing this gap, the study utilizes the resource-dilution perspective to analyze child poverty in Northern Tanzania. The theory posits that households operate with a finite reservoir of resources (Blake, 1981; Downey, 1995). As a family expands or faces economic shocks, these inputs stretch thinner, leaving less support for each child (Kalmijn and van de Werfhorst, 2016; Steelman et al., 2002). Growing family sizes without a matching increase in household assets naturally stretch resources, leaving caregivers struggling to invest adequately in each child's well-being (Svensson and Lindström, 2022). This internal stretching provides a structural explanation for why vulnerable children often face severe, overlapping deprivations across basic needs, rights, and social services (Gordon et al., 2003).

Consequently, poverty interventions must evaluate the broader household ecosystem rather than treating the child in isolation. By applying average indexing to synthesize these concurrent struggles into ordered classifications (Very Poor, Poor, and Not Poor). This study explores how specific socio-economic and demographic factors contribute to child poverty in Northern Tanzania.

## 2 Methodology

### 2.1 Study area

The study was conducted in Moshi District, located in the Kilimanjaro Region of northern Tanzania. This area provides an ideal setting for a sub-national analysis because its overall economic success actually hides deeper, localized struggles. Nationally, children under 18 make up about half of the population, and they account for roughly 35% of the population within the Kilimanjaro Region [National Bureau of Statistics, 2022]. Statistically, Tanzania faces a high national multidimensional child poverty rate of around 74% National Bureau of Statistics (NBS) and United Nations Children's Fund (UNICEF), 2019. Conversely, the Kilimanjaro Region boasts one of the lowest monetary poverty rates in the country at just 4.4% (National Bureau of Statistics, 2019). This low percentage masks real inequalities within the community, making Moshi a compelling place to examine what actually drives child deprivation at the household level.

Families across Moshi District live and work in very different environments, stretching across both the Rural District Council and the Urban Municipal Council. The local economy is a varied mix of farming and non-agricultural work, meaning households differ vastly in their access to stable jobs, marketplaces, and basic social services. These shifting economic realities directly dictate the size of a family's shared resource pool. When households rely heavily on unpredictable subsistence farming or have limited job options, the resulting financial pressure forces caregivers to stretch their finite resources thinnest. This internal stretching directly limits a family's capacity to invest in essential child needs like proper nutrition, healthcare, and education, transforming local economic limitations into direct, measurable forms of child poverty (Very Poor, Poor, and Not Poor).

### 2.2 Sample design and sampling procedure

The study employed a probability-based, multi-stage sampling approach to select children aged 7–17 years from schools in Moshi District, Tanzania. Following procedures similar to the 2019 UNICEF Child Poverty in Tanzania survey (UNICEF and National Bureau of Statistics Tanzania, 2019), the sampling process involved three stages as shown in Figure 1.

**Figure 1:**
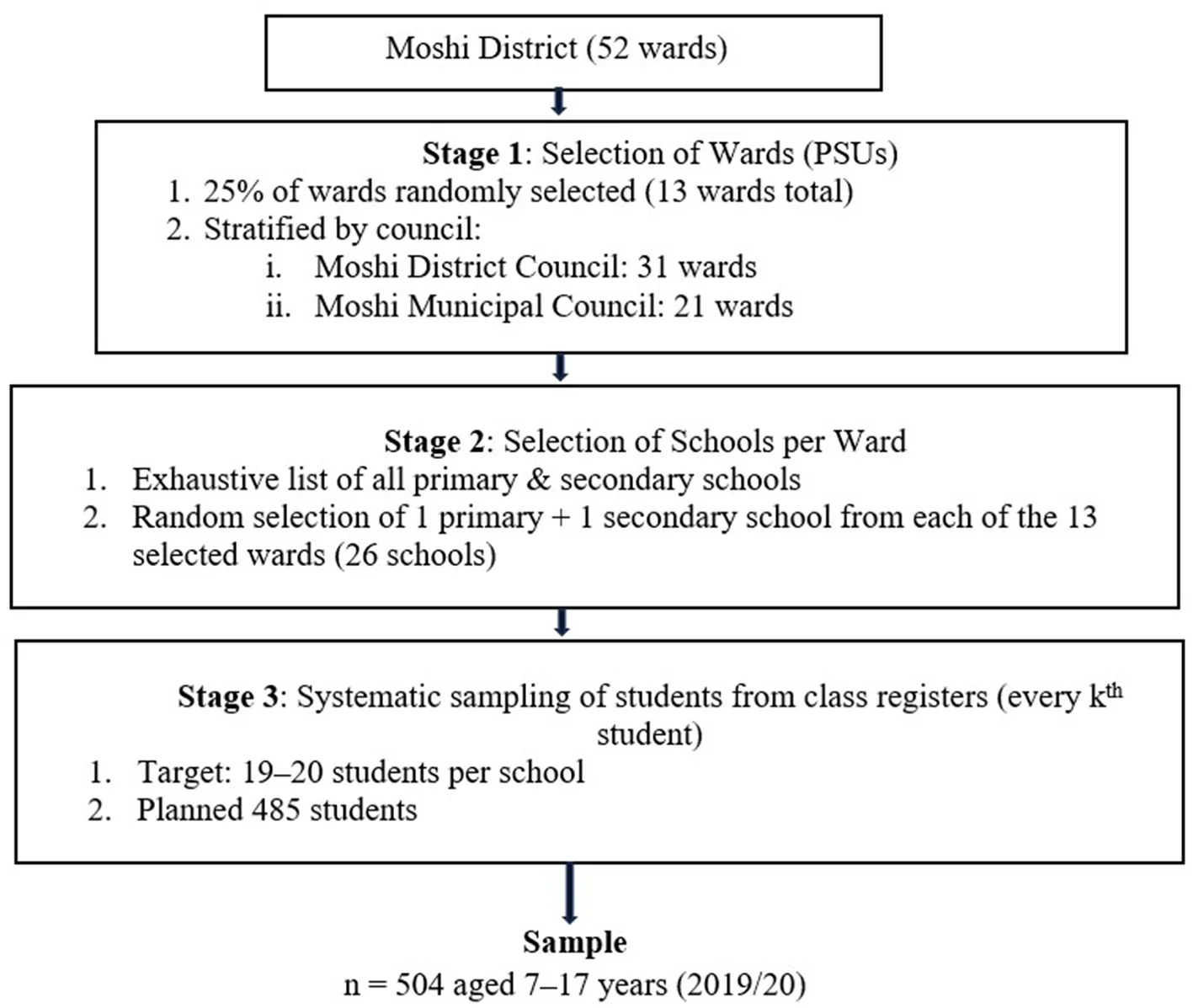
Schematic workflow of the multi-stage sampling procedure.

First stage, a total of 13 wards (25% of the 52 wards) were randomly selected as primary sampling units (PSUs) from the official list provided by district authorities, stratified by the two councils: Moshi District Council (31 wards) and Moshi Municipal Council (21 wards). Thereafter, second stage one primary and one secondary school were randomly selected from the exhaustive list of all schools within the geographic boundaries of each chosen ward. Finally, at the third stage, a systematic random sampling technique was used to select students from school-provided class registers: every *k*th child (where *k* = total eligible students per school/required sample per school, approximately 19–20 per school to reach the overall target) was chosen to ensure proportional representation and minimize bias. This rigorous, multi-stage procedure enhances generalizability while accounting for the district's administrative structure and school distribution. The whole process is shown in the following flow chart.

The sample size was calculated using the single population proportion formula proposed by Cochran (1977) and commonly applied in cross-sectional studies (Lwanga and Lemeshow, 1991):


$$\begin{array}{l} n=p^{*}{\left(1-p\right)}^{*}{\left(\frac{Z}{E}\;\right)}^{2}. \end{array}$$

In this study, *Z* = 1.96 corresponds to a 95% confidence level, *E* = 0.05 represents the desired precision, and *p* = 0.5 was assumed due to the absence of prior population estimates, yielding a conservative sample size. This calculation resulted in an initial minimum sample of 385 children, which was subsequently increased by 20% to account for potential non-response, establishing a calculated operational minimum target of 462 children. However, during field execution, data collection was successfully extended to fully cover all selected administrative household clusters. This transparent tracking sequence yielded a total of 504 valid responses, all of which met the data completeness requirements and were fully retained for final statistical analysis.

Although alternative approaches exist such as power-based or finite population correction formulas, they require prior effect sizes or known population parameters. Given the study's cross-sectional design and objective of estimating population proportions, the Cochran formula was the most appropriate and conservative choice.

### 2.3 Data collection and measurement

A cross-sectional survey design was employed to collect primary data for this study. Child poverty was measured using a multidimensional framework capturing children's access to basic needs, child rights, and essential social services. The basic needs dimension included indicators related to adequate food, clothing, and shelter. The child rights dimension assessed children's ability to express opinions at home and at school, as well as their perceived autonomy in everyday decisions. Access to social services captured availability and use of drinking water, schooling, and health services outside school. Data were collected using structured questionnaires administered to children, with supplementary information obtained from their guardians where necessary. The selection and construction of these indicators draw on established approaches in the child poverty literature (Dinisman and Ben-Arieh, 2016; Haanpää et al., 2019; Basharat and Ambreen, 2019; Aaberge and Brandolini, 2014).

To measure childhood deprivation, this study evaluates three main dimensions using nine indicators: basic needs (food, clothing, and shelter), child rights (attaining personal desires, expressing opinions at home, and expressing opinions at school), and social services (clean water, schooling, and health services) (Basharat and Ambreen, 2019; Dinisman and Ben-Arieh, 2016; Haanpää et al., 2019). The indicators were originally measured on a seven-point Likert scale. To construct the multidimensional poverty variable, these multi-point Likert items were aggregated using an average indexing approach to form a continuous composite index for each child. Thereafter, following the scale reduction protocols established by Al Rubaish (2010, 2011) and Al Rubaish et al. (2012), the continuous median scores across the individual indicators were calculated to group the items cleanly into the three final categories. Fixed cut-off thresholds were then applied to this composite index to segment the sample population into three progressive, rank-ordered categories: Very Poor (representing high deprivation and low average scores), Poor (representing moderate average scores), and Not Poor (representing low deprivation and high average scores).

### 2.4 Ethical considerations

Prior to field implementation, formal ethical clearance and institutional approval were granted by the (Ref. No. STC/2/243/Part III//8). Following this institutional clearance, secondary administrative research permits were obtained from the Regional Commissioner's Office and the Moshi District Executive Director to grant formal entry into the study areas. Because the targeted study population comprised minor children aged 7–17 years, a strict dual-authorization protocol was enforced to protect this vulnerable group. Before interviews began, data collectors gave information forms to parents or legal guardians explaining the study. Written consent was obtained when parents signed and returned these forms to the data collectors. Afterward, age-appropriate assent was secured from the children. Participants and guardians were informed that participation was entirely voluntary and they could withdraw at any time without consequence. To ensure anonymity, data collectors removed all personal identifiers when compiling the dataset, replacing them with unique numerical codes.

### 2.4 Analytical methods

Both descriptive and inferential data analysis were applied in this study. Further, frequency table and percentages table were used for description of data in this study. Additionally, the design-based Rao-Scott adjusted Chi-square test was used to examine the bivariate associations between the categorical variables under the complex survey design. Moreover, the Adjusted Wald F-test was used to test if there is any overall statistical relationship between variables under the complex survey (svy: ologit). Moreover, *F*-test was used to test if there is any statistical relationship between variables. Finally, OLR was the model used when response variable has category which employs ordinal scale (Zuhdi et al., 2017). Therefore, the study used Ordinal Logistic Regression Model (OLR) since the outcome variable was ordinal scale. Further, the study included all independent variables in ORL model because they were important. To examine the factors associated with child poverty status, an ordinal logistic regression model was employed. The cumulative log-odds of a child *i* being classified at or below poverty category *j*


$$\begin{array}{l} logit\;Pr\left(Y_{i}\leq j|x_{1}\ldots x_{k}\right)=\alpha_{j}-\overset{k}{\underset{i=1}{\sum }}\beta_{i}x_{i},\text{ for }j=1,2 \end{array}$$

where:

α_*j*_ represents the threshold (cut point) parameters for categories *j* = 1, 2,

β_*k*_ represents a regression coefficient,

*x*_1_…*x*_*k*_ represents are the explanatory variables for the model,

β_1_…β_*k*_ are corresponding coefficients.

All data were carefully cleaned and coded through checks for missing values, correction of data entry errors, assessment of outliers and implausible values, and verification of internal consistency across variables; statistical analyses were performed using Stata. The analyses accounted for the complex survey design by applying sampling weights, stratification, primary sampling units (PSUs), and finite population corrections.

## 3 Results and discussion

This section presents and discusses the empirical findings of the study. It begins with a description of the socio-economic and demographic characteristics of the sampled children and their households, followed by an examination of the associations between these characteristics and child poverty status. The final subsection presents results from an ordered logistic regression model to identify key predictors of child poverty severity.

### 3.1 Distribution of children by socio-economic, demographic predictors

Evaluating the socio-economic and demographic determinants of child poverty status requires a precise baseline understanding of the study cohort's underlying composition. This section described data directly self-reported by children and some reported with the help of relative/parents. Table 1 summarizes the socio-economic and demographic characteristics of the sampled children and their households. Slightly more than half of the children interviewed were girls (51.7 percent), while boys accounted for 48.3 percent. In terms of age distribution, the majority of children (69.4 percent) were aged between 14 and 17 years, whereas 30.6 percent were aged between 7 and 13 years. Regarding place of residence, most children (69 percent) were drawn from MDC, compared to 31 percent from MMC. This distribution reflects the sampling design and relative population sizes of the study areas. With respect to education, a large proportion of children were enrolled in secondary education (68.4 percent), while 31.6 percent were attending primary school.

**Table 1:** Distribution of children by socio-economic and demographic characteristics.

Variable	Number	Percentage
Age group of a child		
7–13	148	30.6
14–17	336	69.4
Sex of a child		
Boy	234	48.3
Girl	250	51.7
Residence of a child		
MDC	334	69
MMC	150	31
Current level of education of a child		
Primary	153	31.6
Secondary	331	68.4
Sex of the head of the household		
Male	356	73.6
Female	128	26.4
Household type		
One parent	165	34.1
Two parents	319	65.9
Employment sector of Head of household		
Agriculture	169	34.9
Non-agriculture	145	30
Both	153	31.6
Not-employed	17	3.5
Number of children in the household		
One member	32	6.6
Two members	80	16.5
Three members	101	20.9
Four members	113	23.3
Five members	72	14.9
Six plus members	86	17.8

Household characteristics indicate that most children lived in two-parent households (65.9 percent), while 34.1 percent resided in single-parent households. Male-headed households dominated the sample (73.6 percent), compared to female-headed households (26.4 percent). Employment patterns of household heads reveal that agriculture remains a major source of livelihood, with 34.9 percent engaged exclusively in agriculture. About 31.6 percent were involved in both agricultural and non-agricultural activities, while 30.0 percent depended solely on non-agricultural employment. Only a small proportion (3.5 percent) of household heads reported being unemployed. Household size varied considerably. Households with four children were the most common (23.3 percent), followed by those with three children (20.9 percent). Households with six or more children accounted for 17.8 percent, while those with one child were the least common (6.6 percent). These variations are important, as household size is often associated with resource allocation and child welfare outcomes.

#### 3.1.1 Association between child poverty status and socio-demographic characteristics

Before estimating multivariate models, bivariate analyses were conducted to examine the relationship between child poverty status and selected variables shown in Tables 2a–c. Starting with Table 2a the distribution of children was across poverty categories very poor, poor, and not poor by residence, sex, and age group.

**Table 2a:** Association between child poverty status and selected socio-demographic characteristics.

Socio-demographic variables	Child poverty status			Total	*P*-value
	Very poor	Poor	Not poor		
Child residence					
MDC	34 (10.2%)	77 (23.1%)	223 (66.8%)	334 (100%)	<0.000
MMC	9 (6%)	22 (14.7%)	119 (79.3%)	150 (100%)	
Sex of a child					
Boy	23 (9.4%)	48 (19.7%)	169 (70.9%)	234 (100%)	0.3567
Girl	20 (7.9%)	51 (20.1)	179 (72.1%)	250 (100%)	
Age group					
7–13	23 (15.1%)	35 (23.2%)	90 (61.7%)	148 (100%)	<0.000
14–17	20 (5.8%)	64 (18.4%)	252 (75.8%)	336 (100%)	

**Table 2b:** Association between child poverty status and socio-economic characteristics.

Socio-economic variables	Child poverty status			Total	*P*-value
	Very poor	Poor	Not poor		
Child education level					
Primary	23 (14.8%)	36 (23.1%)	94 (62.1%)	153 (100%)	<0.000
Secondary	20 (5.8%)	63 (18.4%)	248 (75.8%)	331 (100%)	
Employment sector of the head of household					
Agriculture	17 (9.8%)	38 (22.3%)	114 (68%)	169 (100%)	<0.000
Non-agriculture	10 (6.5%)	24 (15.6%)	111 (77.9%)	145 (100%)	
Both Agriculture and Non-agriculture	10 (6.5%)	33 (21.4%)	110 (72.2%)	153 (100%)	
Not employed	6 (35.1%)	4 (22.8%)	7 (42.2%)	17 (100%)	

**Table 2c:** Association between child poverty status and household structure characteristics.

Household structure	Child poverty status			Total	*P*-value
	Very poor	Poor	Not poor		
Sex of the head of the household					
Male	33 (9.1%)	62 (16.8%)	261 (74.1%)	356 (100%)	<0.000
Female	10 (7.2%)	37 (28.4%)	81 (64.4%)	128 (100%)	
Type of household					
One parent	19 (10.8%)	42 (25.1%)	104 (64.1%)	165 (100%)	<0.000
Two parents	24 (7.4%)	57 (17.2%)	238 (75.3%)	319 (100%)	
Number of children in a Household					
One	1 (2.7%)	7 (21.6%)	24 (75.73%)	32 (100%)	<0.000
Two	3 (3.3%)	19 (23.1%)	58 (73.6%)	80 (100%)	
Three	7 (7.2%)	16 (15.6%)	78 (77.2%)	101 (100%)	
Four	9 (7.7%)	17 (14.4%)	87 (77.9%)	113 (100%)	
Five	11 (14.4%)	20 (27.5%)	41 (58.1%)	72 (100%)	
Six plus	12 (13.9%)	20 (22.2%)	54 (63.8%)	86 (100%)	

Overall, most children were classified as not poor across all socio-demographic categories. However, notable variations emerge. A higher proportion of children in MDC were classified as very poor (10.2 percent) compared to those in MMC (6.0 percent). Conversely, a larger share of children in MMC (79.3 percent) were not poor compared to MDC (66.8 percent). The association between place of residence and child poverty status was statistically significant (*p* < 0.001) (Brooks-Gunn and Duncan, 1997). The finding exposes deep regional inequalities in the distribution of deprivation. Consequently, the empirical evidence implies that centralized, homogenous interventions are structurally inadequate. To rectify existing spatial imbalances, social protection frameworks must be geographically targeted and strategically intensified within highly deprived clusters (Mínguez, 2023).

Regarding age-specific vulnerabilities, age dynamics were highly pronounced. Children aged 7–13 years recorded significantly higher proportions of both very poor (15.1%) and poor (23.2%) status compared to those aged 14–17 years (*p* < 0.001). This result is crucial because it disrupts the conventional narrative that older children incur greater economic costs, proving instead that younger children face the highest concentration of poverty (Roelen, 2019). The developmental implication is that household financial strain peaks during middle childhood—a critical period when older siblings are traditionally socialized to guide younger ones toward future opportunities (Bywaters et al., 2016). State support should therefore be front-loaded to families with children under 14 to mitigate long-term developmental harm.

Finally, in evaluating potential gendered impacts, disparities by sex were modest and statistically non-significant (*p* = 0.357). Boys exhibited a slightly higher rate of very poor status (9.4% vs. 7.9%), while girls were marginally higher in the poor category (20.1% vs. 19.7%) (Vukosavljevic et al., 2025). This lack of significance is highly consequential; it implies that despite literature emphasizing how structured African cultural gender roles and biological differences worsen poverty for girls, deprivation in this sample operates as a generalized household affliction rather than a gender-segregated one (Anyanwu, 2014). The actionable implication for stakeholders is that immediate relief funds should prioritize blanket household economic empowerment rather than gender-exclusive targeting, as both sexes experience systemic poverty at statistically identical rates.

Table 2b looks at socio-economic sides, like school level and the household head's occupation. Most children weren't poor, but trends disclosed. Primary students dealt with more tough times 14.8% very poor, 23.1% poor than secondary ones (5.8%, 18.4%, respectively). This strong correlation (*p* < 0.001) echoes findings by Ferguson et al. (2007), which highlight how poverty can hinder early development and trap children in a cycle where they lack the skills needed to move forward (Engle and Black, 2008). Ultimately, these results underscore the vital link between a child's background and their future opportunities. They suggest that by prioritizing universal education and family support, we can disrupt the cycle of poverty and pave the way for better career prospects and long-term wellbeing.

Employment status of the household head emerged as a strong determinant of child poverty. Children from households with unemployed heads faced the highest risk of poverty, with 35.1 percent classified as very poor and only 42.2 percent considered not poor. In contrast, children from non-agricultural households recorded the highest proportion of not poor status (77.9 percent), followed by those engaged in both agricultural and non-agricultural activities (72.2 percent) (Bliznashka et al., 2023; Bai et al., 2023). These differences were statistically significant (*p* < 0.001), underscoring the importance of stable and diversified livelihood sources. Also, the study calls a need for the training to non-farm jobs to drop child poverty and spark growth.

Table 2c explores how household dynamics specifically the sex head of household and family structure shape economic outcomes. Most households in the study were not living in poverty, but the data reveals existing disparities. Children in female-headed households were significantly more likely to experience poverty (28.4%) than those in male-headed households (16.8%). This gap widened in single-parent homes, where rates of extreme poverty (10.8%) and general poverty (25.1%) far outpaced those of two-parent families. These findings, concur with broader research (Sharma, 2023; Brady et al., 2024; Choi et al., 2022; Igandu et al., 2023), highlight a critical vulnerability. It is clear that single mothers face unique hurdles in providing for their families, signaling a profound need for supportive policies and community-based initiatives, especially within developing nations.

Further, Number of children in a household has a strong relationship to child poverty. Poverty levels increased with the number of children in the household. One child household spot had nearly zero very poor, but five-plus spiked very poor and poor. Significant (*p* < 0.001). This result spotlights population pressure which has implication to household and community resources utilization and also the issues of family planning policy to reduce child poverty (Krishnaji, 1980).

#### 3.1.2 Socio-economic, demographic factors, and child poverty status

This subsection explores how socio-economic and demographic factors come together to affect child poverty in real, everyday ways. Using an ordered logistic regression model, the study identifies key household and child characteristics that influence whether children move out of poverty categories. Building on the earlier descriptive and bivariate overviews, it highlights broader structural issues like family income sources, population pressures, and opportunities for growth—elements central to modern development research that focus on group dynamics over individual traits as shown in Table 3.

**Table 3:** Ordered logistic regression estimates of socio-economic and demographic determinants of child poverty status.

Predictor variable	Adjusted odds ratio (AOR)	95% CI	*p*-value
Age group of a child (Ref: 7–13 years)			
14–17 years	2.09	1.47 – 2.96	**< 0.001**
Sex of a child (Ref: Boy)			
Girl	1.08	0.92 – 1.28	0.347
Residence of a child (Ref: MDC)			
MMC	1.64	1.26 – 2.12	**< 0.001**
Current Education level of a child (Ref: Primary)			
Secondary	0.99	0.69 – 1.42	0.949
Sex of H/H (Ref: Male)			
Female	0.76	0.60 – 0.97	0.027
Household type (Ref: One-parent)			
Two-parent	1.37	1.14 – 1.66	**0.001**
Employment sector of H/H (Ref: Agriculture)			
Non-agriculture	1.52	1.20 – 1.92	**0.001**
Both Agriculture and Non-agriculture	1.10	0.91 – 1.33	0.31
Not employed	0.23	0.14 – 0.40	**< 0.001**
Household size (Ref: one member)			
Two members	0.78	0.49 – 1.25	0.288
Three members	0.90	0.55 – 1.49	0.686
Four members	0.98	0.60 – 1.59	0.918
Five members	0.42	0.26 – 0.66	**< 0.001**
Six plus members	0.44	0.28 – 0.70	**0.001**
/cut1	−1.93	[−2.43 – 1.44]	
/cut2	−0.37	[−0.87 – 0.13]	

Bold values show results that are statistically significant at the 0.1% level (*p* ≤ 0.001).

The regression model reveals that the selected covariates are jointly and highly statistically significant [*F*_(14,29)_ = 23.42, *p* < 0.001], affirming that observable socio-economic and demographic traits meaningfully account for variations in child poverty, far beyond mere chance. This result highlights actionable levers for policy intervention, underscoring that poverty is not an inevitable fate but a product of modifiable circumstances. The multivariate findings echo and refine the bivariate patterns, illuminating the comparative influence of predictors while adjusting for confounders.

The age of a child fundamentally shapes their experience with poverty. Study findings reveal that Adolescents aged 14–17 had 2.09 times higher odds of being in a less severe poverty category compared to younger children (AOR = 2.09; 95% CI: 1.47–2.96; *p* < 0.001). This shift likely reflects the expanding roles older children play: as they mature, they often contribute more to household productivity, navigate wider social circles for support, or command a larger share of scarce family resources. This pattern mirrors the work of Haanpää et al. (2019), who noted that poverty risk tends to recede as children grow. Ultimately, these results suggest that for interventions to be effective, they must evolve alongside the child, providing a safety net that adapts to the unique pressures of each developmental stage.

Household composition was significantly associated with multidimensional child poverty, suggesting that family demographic structure is an important correlate of child deprivation. Children living in two-parent homes showed higher adjusted odds of being classified as non-poor compared to those in single-parent settings (AOR = 1.37; 95% CI: 1.14–1.66; *p* = 0.001). These findings support the idea that dual-parent families can pool their incomes and share daily caregiving tasks, which provides a stronger financial cushion for children. This pattern aligns with Mood and Jonsson (2016), who point out that single-parent families face much higher risks of material hardship due to limited earning capacity. Ultimately, these results highlight how a stable, two-parent family ecosystem helps protect children from severe deprivation, emphasizing the need for targeted social policies to support single-parent households.

Household livelihood strategies fundamentally shape child wellbeing and domestic resource distribution. Children in non-farming households were significantly more likely to fall into less severe poverty categories than those from families dependent on agriculture (AOR = 1.52; 95% CI: 1.20–1.92; *p* = 0.001). Rather than farming being inherently poverty-producing, this gap reflects the structural and climate challenges of subsistence agriculture in Northern Tanzania, including unpredictable weather and seasonal economic shocks. Non-farming jobs, by contrast, offer a stable income that insulates the household budget (Gordon et al., 2003). The critical role of reliable earnings is underscored by employment status: if the household head is unemployed, a child's chances of escaping severe poverty drop drastically (AOR = 0.23; 95% CI: 0.14–0.40; *p* < 0.001). These findings align with Mulugeta et al. (2017), confirming that off-farm diversification is a powerful pathway for rural poverty alleviation. From a resource-dilution perspective, stable employment expands the family's total resource pool and buffers them against localized shocks (Kalmijn and van de Werfhorst, 2016). Caregivers who secure a steady flow of income can prevent domestic resources from stretching too thin, allowing for adequate investment in children's growth, education, and nutrition (Blake, 1981; Downey, 1995).

Family size fundamentally structures child wellbeing. While households with two to four members show no statistically significant differences from single-person configurations, the proportion of children classified as very poor increased markedly among households with five or more members. Specifically, children in households with five members (AOR = 0.42, 95% CI: 0.26–0.66, and *p* < 0.001) or six-plus (AOR = 0.44, 95% CI: 0.28–0.70, and *p* = 0.001) had significantly lower odds of being classified in a less severe poverty category (Blake, 1981; Downey, 1995). This pattern confirms that expanding family sizes stretch finite resources thin, forcing households to reduce expenditures on vital child-level needs like nutrition, education, and healthcare (Gordon et al., 2003; Kalmijn and van de Werfhorst, 2016). These outcomes align with Newhouse et al. (2016), who demonstrated a robust link between large households and severe child deprivation, highlighting the critical need for localized family planning and targeted social support systems.

Sex of household head the household remains a significant predictor of child deprivation, even when controlling for other socioeconomic characteristics. Children residing in female-headed households exhibited significantly lower odds of being classified in a less severe poverty category compared to those in male-headed homes (AOR = 0.76, 95% CI: 0.60–0.97, and *p* = 0.027). This differs from some studies like Haanpää et al. (2019) that saw benefits in female-headed families, possibly due to varying local supports. It probably reflects deeper inequalities in jobs, property, and loans for women, suggesting a need for policies that boost women's opportunities and fairness.

Further, found that girls had slightly higher odds of experiencing multidimensional poverty than boys, although the child's sex was not significantly associated with multidimensional poverty (AOR = 1.08, 95% CI: 0.92–1.28, and *p* = 0.347). This suggests that household-level deprivation may have a stronger influence than individual characteristics such as sex, as limited household resources and living conditions can affect both boys and girls similarly. Similarly, school level was not significantly associated with multidimensional poverty (AOR = 0.99, 95% CI: 0.69–1.42, and *p* = 0.949). This contrasts with Haanpää et al. (2019) finding lower grades tied to less poverty. In this setting, the findings suggest that household poverty may have a stronger influence than schooling, limiting children's choices, participation, and opportunities regardless of their education level. This points to the need for broader interventions that address household deprivation and support children's wellbeing.

## 4 Conclusion and recommendations

### 4.1 Conclusion

This study demonstrates that child poverty in Northern Tanzania is inextricably linked to the broader demographic profiles and economic constraints of the domestic unit. Specifically, findings indicates that age of a child, residence, expanded families size, high dependence ratio and unstable household employment significantly escalate a child's vulnerability to deprivation. Children residing in smaller, economically active households are substantially less likely to experience severe deprivations, whereas those in expanding households remain highly vulnerable. Framed within the sociology perspective, the findings confirm that child wellbeing dictated by internal resources allocation dynamics and family economic security. Because large family configurations structurally dilute available household resources, sustainable poverty reduction cannot be achieved through isolated, individual-centric child welfare models. Instead, interventions must comprehensively target the holistic socio-economic environment of the entire family ecosystem.

### 4.2 Recommendations

Based on the empirical evidence, this study offers coordinated policy recommendations to enhance family wellbeing and achieve sustainable reductions in multidimensional child poverty.

First, to safeguard household-level economic stability, public policy must prioritize structural livelihood diversification beyond traditional subsistence agriculture. This requires expanding non-farm employment opportunities, investing in localized vocational training, and establishing entrepreneurial support systems, with explicit prioritization given to households headed by unemployed individuals.

Second, targeted social protection mechanisms must be deployed to high-dependency households. Directing conditional or unconditional social safety nets toward families with a high number of dependents will buffer their immediate capacity to meet children's fundamental needs, including nutrition, healthcare, safe shelter, and education.

Third, structural access to family planning education and reproductive health resources must be expanded. Helping households make informed fertility decisions directly mitigates domestic resource dilution and strengthens the structural caregiving capacity of families to align with their available economic resources.

Finally, macro-level structural adjustments must be paired with community-based programs that systematically integrate children's voices into policy design. Providing opportunities for children to express their lived experiences ensures that localized interventions remain responsive and inclusive. Ultimately, a synchronized strategy that combines livelihood fortification, targeted social safety nets, reproductive health advocacy, and child participation offers the most viable path to breaking the cycle of child poverty in Northern Tanzania.

### 4.3 Limitations and future research directions

This study has a few clear limitations that open up interesting paths for future research. First, the cross-sectional data from Moshi District only gives us a localized snapshot, meaning the findings cannot be easily applied to other parts of Tanzania with different socio-economic setups. Future studies should use longitudinal designs over wider geographic areas to track how child poverty and family structures actually change over time.

Second, a major structural limitation is the lack of data on land tenure and farm size. In agrarian areas like Northern Tanzania, secure property rights and plot sizes heavily dictate a household's wealth and long-term stability. Without this fieldwork data, our model could not evaluate how land rights or land fragmentation affect child poverty. Future research should include land registration, farm acreage, and crop commodity data to show how property dynamics influence family vulnerability.

Finally, the study did not directly measure household proximity to regional economic drivers like agro-industries or tourism. To keep our policy conclusions strictly data-driven, these broader macroeconomic factors were left out of the regression models. Future research should look at how these macro-level job markets interact with internal family resource sharing to map out clearer paths for reducing child deprivation.

## Data Availability

The raw data supporting the conclusions of this article will be made available by the authors, without undue reservation.
